# Classification of pain expression images in elderly with hip fractures based on improved ResNet50 network

**DOI:** 10.3389/fmed.2024.1421800

**Published:** 2024-07-01

**Authors:** Yang Shuang, Gong Liangbo, Zhao Huiwen, Liu Jing, Chen Xiaoying, Shen Siyi, Zhu Xiaoya, Luo Wen

**Affiliations:** ^1^The 2nd Ward of Hip Joint Surgery, Tianjin Hospital, Tianjin, China; ^2^College of Information Technology and Engineering, Tianjin University of Technology and Education, Tianjin, China; ^3^The 2nd Ward of Joint Surgery, Tianjin Hospital, Tianjin, China; ^4^Traumatic Orthopedics Department, The 3rd Ward of Hip Joint Surgery, Tianjin Hospital, Tianjin, China; ^5^The 2nd Ward of Knee Trauma Department, Tianjin Hospital, Tianjin, China

**Keywords:** expression recognition, Resnet50 network, MTCNN face detection, Bayesian optimization, pain assessment

## Abstract

The aim of this study is designed an improved ResNet 50 network to achieve automatic classification model for pain expressions by elderly patients with hip fractures. This study built a dataset by combining the advantages of deep learning in image recognition, using a hybrid of the Multi-Task Cascaded Convolutional Neural Networks (MTCNN). Based on ResNet50 network framework utilized transfer learning to implement model function. This study performed the hyperparameters by Bayesian optimization in the learning process. This study calculated intraclass correlation between visual analog scale scores provided by clinicians independently and those provided by pain expression evaluation assistant(PEEA). The automatic pain expression recognition model in elderly patients with hip fractures, which constructed using the algorithm. The accuracy achieved 99.6% on the training set, 98.7% on the validation set, and 98.2% on the test set. The substantial kappa coefficient of 0.683 confirmed the efficacy of PEEA in clinic. This study demonstrates that the improved ResNet50 network can be used to construct an automatic pain expression recognition model for elderly patients with hip fractures, which has higher accuracy.

## 1 Introduction

Pain is often accompanied by changes in behavior, including facial expressions that have received significant attention (1). Similar to expressions of emotion (2), facial expressions during pain play a critical role in communicating information about the experience (3). Hip fracture is a common orthopedic injury that exponentially increases in incidence with age (4). Intense preoperative pain after hip fractures is common and can lead to long-term effects, such as residual pain and poor joint function (5). Accurate detection and estimation of pain is essential for pain management, such as whether and at what level analgesics are administered to the patient. Sometimes the pain on the patient’s face is much worse than it really is (6), they are less accurately detected than the six basic emotions and are often mistaken for negative emotions characterized by similar facial muscle movements, such as disgust (7).

Facial expression of pain is one potential method for improving the detection and estimation of pain. It is the most salient form of pain behavior and communicates information about the sensory and affective components of the multidimensional experience of pain (8). Evidence suggests that the core expression of pain is characterized by four facial muscle movements, including brow-lowering, orbit tightening, levator-contraction, and to a lesser extent, eye closure (9). Facial expressions of pain are associated with different processes than self-report and provide complementary information that may be more valid in some circumstances, such as among individuals with cognitive or expressive impairments (10).

Face recognition is the process by which a computer determines an individual’s identification based on their facial features. Traditional face recognition technology first calculates the feature description factors describing each individual’s identity based on the distribution of each pixel in the face image (11). The field of face recognition has advanced significantly with the development of deep learning, which has increased the efficiency and precision of face recognition compared to traditional techniques. The primary benefit of deep learning is its ability to train on enormous amounts of data, gradually adapt to various scenarios, and discover the optimal features to represent the data (12). Convolutional neural networks (CNN) and their variant networks have excellent effects on image processing, laying the foundation for the application of deep learning in the recognition of pain expression images of elderly individuals with hip fractures (13).

This study selected the ResNet50 network in this study based on the performance of different networks in image recognition projects through literature review, especially compared with VGNet19 and DenseNet121, which has best training time and memory performance (14). ResNet has been known by winning the ImageNet Large Scale Visual Recognition Challenge (ILSVRC) in 2015, which perfectly solves the problem of gradient vanishing in deep neural networks. To improve the detection, recognition, and classification results, the ResNet50 network model has been improved by MTCNN network and Bayesian Optimization, reducing manual facial feature extraction steps, to minimize dependencies for manual experience and expert knowledge.

To improve the efficiency and accuracy of pain assessment, we intend to construct a facial recognition model of pain expression for elderly patients with hip fractures through deep learning technology. To address the issues of determining network structure, excessive number of training runs, and excessive time for processing facial expression classification in convolutional neural networks, we will design an improved ResNet50 network based on Bayesian optimization, and use this algorithm to construct an automatic classification model of pain expressions in elderly patients with hip fractures.

## 2 Methods

### 2.1 Technical route

**Figure 1:**
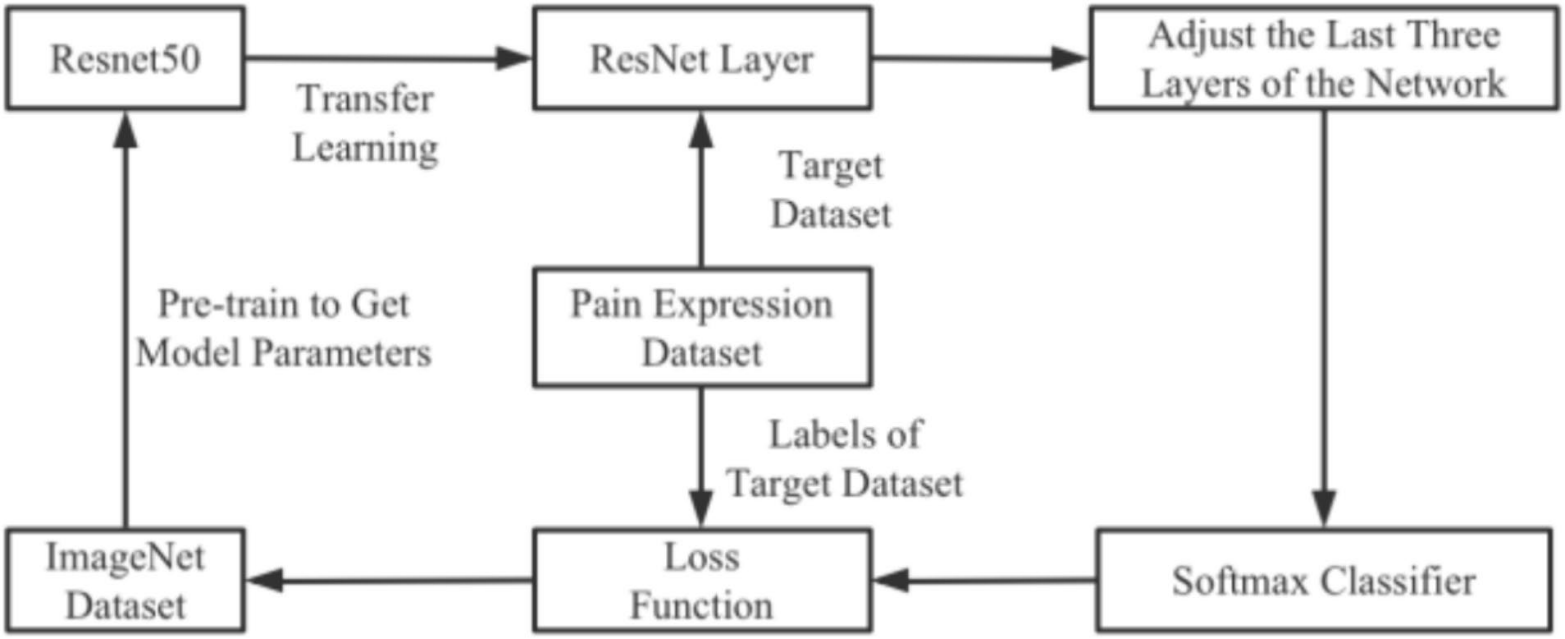
Flow chart of pain expression recognition method.

The first phase proposed an automatic image classification model for pain expression based on the ResNet50 network. This approach addressed the shortcomings of traditional machine learning in facial expression image recognition, the high complexity of manual feature extraction and the limitations of conventional deep learning, such as network degradation with increasing network depth. The model flow chart depicted in Figure 1. Face detection was achieved by video using Multi-Task Cascaded Convolutional Neural Networks (MTCNN). Detected pre-processed images were inputted into the ResNet50 network, which was trained and optimized for pain grade classification using Bayesian optimization. Once the requirements were met, voice playback and data recording functions has been implemented. The Pain Expression Evaluation Assistant (PEEA) is an automated software system that analyzes facial pain expressions for the detection and classification of features relevant to pain assessment.

### 2.2 Multi-task cascaded convolutional neural networks

Traditional facial detection techniques used Adaboost facial detection algorithm and Active Appearance Model (AAM). AAM added facial texture information to the shape information of facial images by using 66 facial feature markers to detect the global information of facial images. However, AAM required manual labeling of facial feature points, making the model construction process cumbersome and complex, which has poor generalization performance. The Adaboost facial detection algorithm combined a large number of weak classifiers with average classification ability to form a strong classifier (15). A weak classifier compared by different Haar Like features to select the classifier with better features. Adaboost weighted and combined these weak classifiers to obtain a strong classifier. The Adaboost algorithm obtained weak global features, which has poor generalization ability, especially for specific populations, leading to low recognition accuracy.

In engineering practice, the MTCNN algorithm is renowned for its high detection speed and accuracy. MTCNN employs a three-layer cascade architecture to accomplish face detection and key point localization in images (16). MTCNN comprised three networks: P-Net, R-Net and O-Net.

The three stages of MTCNN can be simply explained as follows: The first network layer: P-Net, which scaled the image at multiple levels and performed sliding detection on each image using a 12 × 12 sliding window with a step size of 2 for each scale. Small pictures can detected large faces, while large pictures can detected small faces. All detected face frames undergo NMS (Non-Maximum Suppression) to obtain the face frames, which were then converted to the original size, and the short side was filled to convert them into squares of 24 × 24. The second network layer: R-Net, which processed multiple 24 × 24 faces from the previous step using the CNN network to obtain more precise face frames, which then undergone NMS and ultimately re-sized to a 48 × 48 square. The third network layer: O-Net, which take multiple 48 × 48 faces from the previous step as input to obtain multiple more accurate boxes, five face position points and confidence scores. NMS is then applied to the face frames to obtain the final required face frame.

The input image of MTCNN has not limited by size. By reducing the size of the convolution kernel, the computational complexity and weight parameters have reduced, and by optimizing the activation function, the network performance has significantly improved. Therefore, this study choose the MTCNN facial detection algorithm to extract facial expressions.

### 2.3 ResNet 50 convolutional neural network

A transfer learning approach utilizing ResNet50 has been employed in this study. The method involved pre-training a convolutional neural network (CNN) on a large existing dataset, and then transferring the pre-trained CNN to a target dataset for fine-tuning. The proposed model has been initially pre-trained on the ImageNet dataset (17). All layers, except the Softmax layer, are initialized with the pre-trained model parameters, as opposed to traditional random initialization (18). The Softmax layer is added to process the dataset used in this study. This transfer learning method offered several advantages, including superior model generalization performance, significant depth, high accuracy and good convergence (19).

**Figure 2:**
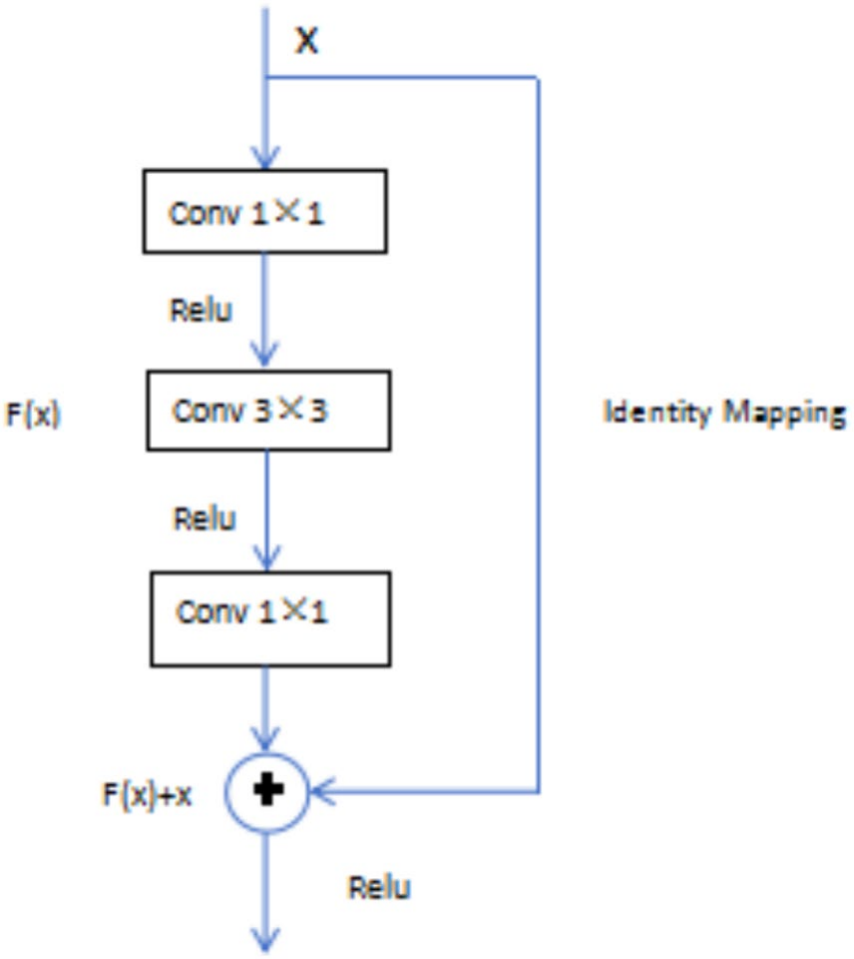
Residual block structure.

The ResNet 50 model introduced residual blocks in deep neural networks, perfectly solving the problem of “gradient vanishing.” The basic idea of this network was “Identity Mapping,” which has mean that the output of each layer was the same, and residual mapping has easier to optimize than the original mapping, reducing the computational burden of the network (18). In the residual block, assuming *x* was the input of the model, *H(x)* was the output of the residual network, and the output after convolution operation was *F(x)*, then *H(x) = F(x) + x*. As long as *F(x) = 0*, which formed the identity mapping function *H(x) = x* mentioned earlier, transforming the problem into an easily fitting residual function *F(x) = H(x)−x* (Figure 2).

**Figure 3:**
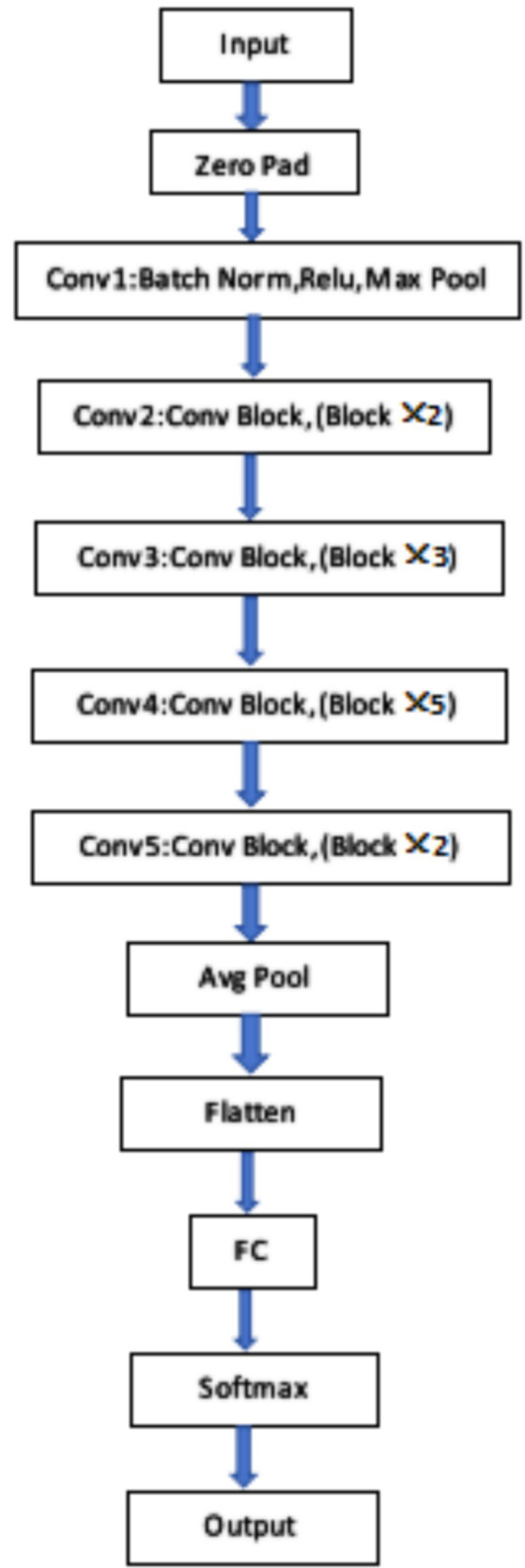
ResNet50 structure.

The ResNet50 network consisted of 49 convolutional layers and 1 fully connected layer, with a total of 5 convolutional operation stages. The image entered the first stage of convolution, which has been batch regularization, activation function, and max pooling operation. After that, the network entered the second stage of convolution. From the second stage to the fifth stage of convolution, residual blocks with dimensions has been added, and each residual block contained three convolutional layers. After all convolution operations, the final input has into the fully connected layer and the corresponding classification has output through the softmax layer, as shown in Figure 3.

### 2.4 Bayesian optimization

Bayesian optimization is an algorithm, which is used Bayesian probability to search for the optimal value of an objective function, which the probabilistic surrogate model and acquisition function were the key components (20). The most widely used probabilistic surrogate model was the Gaussian process, and the acquisition function was based on the posterior probability of the objective function. The goal of the Bayesian optimization algorithm was to minimize the total loss *r*, and which is achieved by selecting the evaluation point *x_i_* using the acquisition function, which is formulated as follows Equation (1):


(1) $$\begin{array}{l} x_{i+1}=\max_{x\in X}\lambda \left(x,D_{1:i}\right)\\ r_{i}=\left|y^{*}-y_{i}\right| \end{array}$$

where X is the decision space, λ(x,D_1_:i) is the acquisition function, and y* is the optimal solution (21).

The implementation of Bayesian optimization followed the procedure outlined below (22):

Determining the maximum number of iterations *N*.

Using the acquisition function to obtain the evaluation point *x_i_.*

Evaluating the acquisition function *y_i_* at the evaluation point *y_i_.*

Updating the probabilistic agent model by integrating the data *D_t_*.

If the previous number of iterations *n* is less than the maximum number of iterations *N*, output *x_i_*; otherwise, return and continue iterating, until output *x_i_*.

## 3 Experiment

### 3.1 Database construction

In order to construct the pain expression database, this study followed the existing methods and schemes. Firstly, the researchers recorded videos of elderly patients with hip fractures expressing different degrees of pain. Secondly, key frames capturing the required expressions were extracted from the videos. Experienced orthopedic specialist clinicians evaluated and classified these key frames. Thirdly, consistent images were selected and included in the database.

Patients with hip fractures attending the inpatient facility of a hospital’s hip joint trauma department participated in the video collection. The Hospital Research Ethics Committee approved the study protocol, and patients provided the informed consent before participation. The research objective, subjects’ rights and investigators’ obligations were explained to the respondents using uniform language. The privacy of the participants was ensured throughout the study.

For image or video detection, a clinician used a camera to capture the patient’s facial expressions. The camera was positioned 1–1.5 meters away from the patient’s face, and the video acquisition time was 20–25 s. During this period, the patient did not need to hide their state.

The inclusion criteria for the study were: (1) the elderly has been diagnosed hip fractures according to radio-logical diagnostic criteria (23); (2) patients were entirely awake who have understanding independently; (3) age ≥ 65; (4) patient agrees to participate in this study. The exclusion criteria were: (1) patient has neurological diseases, such as Alzheimer’s disease; (2) patient has deafness, aphasia and other symptoms that hindered communication.

The method involved sequentially recording videos of elderly individuals expressing different degrees of pain. The required key expression frames were extracted from these videos, and experienced clinicians evaluated and classified them. The acquired images were labeled according to the pain grade of mild, obvious, severe, and intense. To ensure the reliability of the database, images with high consistency in multiple evaluators’ scores have mainly selected and included in the database, which summarized in the following aspects: turning over one the bed, moving from flat car to bed, lower limb flexion and extension training, straight leg raising training and bedside standing.

To focus on facial expression recognition algorithm research and minimize the influence of other factors, the original images were being normalized. The method involved three steps: rotation correction, image cropping and scale normalization. The purpose was to correct for background interference and angle offsets caused by the shooting environment and changes in face pose. This ensured the face of the patient in the image was upright and both eyes were in a horizontal state. The method removed redundant background information as much as possible, retaining only the effective facial region containing expressions. The center points of the two eyes were manually marked, and the image was rotated using the axis connecting the center points of the eyes as the reference. The center points of the eyes were adjusted to the same horizontal line to eliminate angle deviation. Next the patient’s facial region was manually cropped from the corrected image. Researchers performed pain classification on facial expression images after data augmentation using the Prkachin & Solomon pain severity method. They discovered and confirmed that the four movements of eyebrow depression and convergence (AU4), eye socket tightening (AU6 and AU7), levator muscle contraction (AU9 and AU10), and eye closure (AU43), which can carry a lot information about pain. The pain grade can be evaluated by evaluating the severity of AU, which is the Prkacin & Solomon pain grade, the expressed as Equation (2):


(2) $$\begin{array}{l} \mathrm{PSPI}=\mathrm{AU}4+\max\left(\mathrm{AU}6,\;\mathrm{AU}7\right)+\\ \;\max\left(\mathrm{AU}9,\;\mathrm{AU}10\right)+\mathrm{AU}43 \end{array}$$

**Figure 4:**
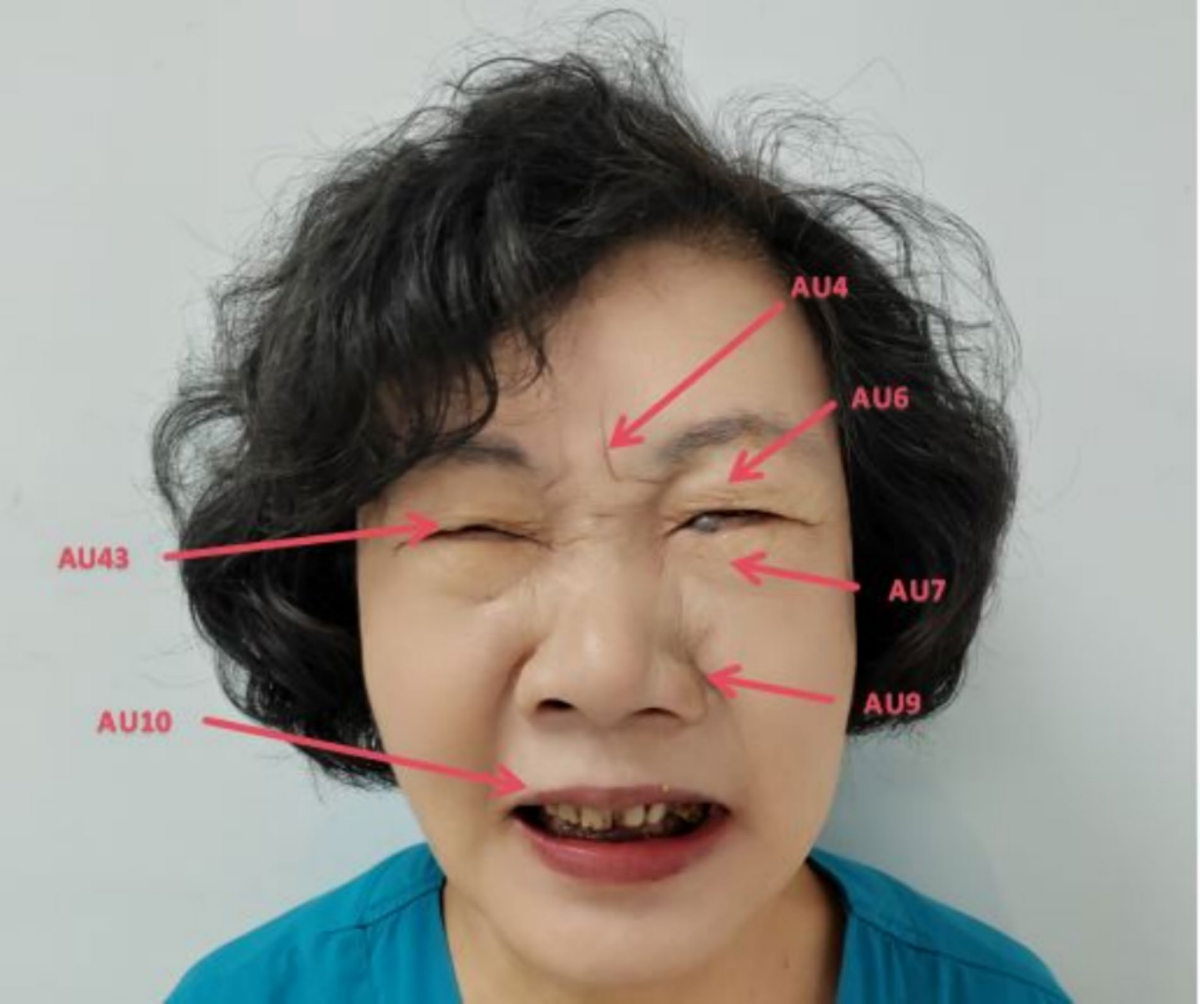
The core facial movements of pain expression.

The total pain grade is the sum from AU4, the maximum intensity of AU6 and AU7, the maximum intensity of AU9 and AU10, and AU43 (taken as 0 or 1) (24). The PSPI pain measurement standard is the only measured method in image frames, which can be used to evaluate the degree of pain currently. The PSPI scoring system used the Likert 5-level scoring method (a-e), which the score range of each item has from 0 to 4. Higher score means the patient has stronger pain symptoms. The core facial movements of pain expression has been shown in Figure 4 (25).

The score of Figure 4 as follow Equation (3):


(3) $$\begin{array}{l} \mathrm{PSPI}=4e+\max\left(6d,7e\right)+\max\left(9c,10d\right)+43b\\ =4+\max\left(3,2\right)+\max\left(4,3\right)+1\\ =4+3+4+1\\ =12 \end{array}$$

The evaluation team involved an anesthesiologist, an orthopedic surgeon and two clinical nurses, who assessed the collected facial pain expression images by PSPI. All staff members had over 10 years experience in clinical work. After evaluating and selecting from the video key frames, a database of facial pain expressions of elderly patients has been established in accordance with guidelines for pain management (1). The dataset consisted of 4,538 images, which included 2,247 images of mild pain (VAS: 1–3), 735 images of obvious pain (VAS: 4–6), 729 images of severe pain (VAS: 7–8) and 827 images of intense pain (VAS: 9–10).

The entire dataset has been randomizing, which was partitioned into three sets: the training set (60%), validation set (20%), and test set (20%). The training dataset underwent rotating, cropping and translating to increase the number of training samples, which improved the robustness and generalization performance of model.

In the field of computer vision, the model’s performance depended on not only the data and the quality of its structure but also the optimizer, loss function and data enhancement methods. The effectiveness of the model’s training strategy, such as the optimizer, data augmentation and regularization technique, which impacted its performance importantly and significantly. This study utilized data enhancement techniques (Table 1) to improve the accuracy of model training.

**Table 1:** Comparison of model accuracy before and after data enhancement.

	Accuracy/%
Before data enhancement	67.62
After data enhancement	73.99

Furthermore, Bayesian optimization was employed to determine the optimal hyperparameters, including the learning rate with a maximum of 60 iterations, which was expected to enhance the acquisition function. Table 2 showed the decision space for the optimization process.

**Table 2:** Super parameters and their ranges of improved ResNet50.

Hyperparameter	Minimum value	Maximum value
Initial learn rate	1 × 10^−2^	1
Momentum	0.8	0.98
L2 Regularization	1 × 10^−10^	1 × 10^−2^
Optimizer	RMSprop, Adam,SGDM	

In this study, the classification and generalization ability of the model for recognizing pain expressions in elderly patients with hip fracture were assessed based on the prediction accuracy and model training time (26).The formula used for the prediction accuracy *P_1_* as follow Equation (4):

(4) $$p1=\frac{mTA}{nT}\times 100\%$$

Where *n_T_* is the number of test sets used to validate the model, and *m_TA_* is the number of accurately classified samples in the sample test set.

### 3.2 Pain grade prediction

Inputting the normalized image into the trained improved Resnet50 model, using the Softmax classifier to receive the feature matrix input by the fully connected layer, and outputting the probability value of each category corresponding to the input object, assuming that there are *N* input objects ${\left\{X_{i}Y_{i}\right\}}_{i-1}^{k}$, the label for each object is $y_{i}\in \left\{1,2,\ldots ,k\right\}$, which is the number of the model output categoriesk≥2(20), 4 grade classification (1, 2, 3, 4),k is performed for the pain expression, and maximum value is 4. Input $X_{i}$, using the assumption function $f_{\theta }\left(X_{i}\right)$to estimate the probability value of its corresponding class *j*: $P\left(y=j/X_{i}\right)$, and the function is Equation (5):


(5) $$f_{\theta }\left(x_{i}\right)=\left[\begin{array}{l} P\left(y_{i}=1|x_{i};\theta \right)\\ P\left(y_{i}=2|x_{i};\theta \right)\\ \cdot \\ \cdot \\ \cdot \\ P\left(y_{i}=k|x_{i};\theta \right) \end{array}\right]=\frac{1}{\overset{k}{\underset{j-1}{\sum }}e^{\theta_{j}^{T}x_{i}}}\left[\begin{array}{l} e^{\theta_{1}^{T}x_{i}}\\ e^{\theta_{2}^{T}x_{i}}\\ \cdot \\ \cdot \\ \cdot \\ e^{\theta_{k}^{T}x_{i}} \end{array}\right]$$

The loss function for the Softmax classifier is (19) Equation (6):


(6) $$J\left(x,y,\theta \right)=-\frac{1}{N}\left[\overset{N}{\underset{i-1}{\sum }}\overset{k}{\underset{j-1}{\sum }}1\left\{y_{i}=j\right\}\log_{2}\left(\frac{e^{\theta_{j}^{T}x_{i}}}{\overset{k}{\underset{j-1}{\sum }}e^{\theta_{j}^{T}x_{i}}}\right)\right]$$

**Figure 5:**
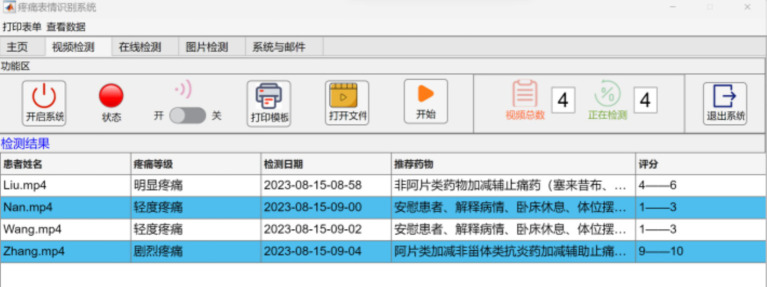
Patients’ video pain assessment result output.

**Figure 6:**
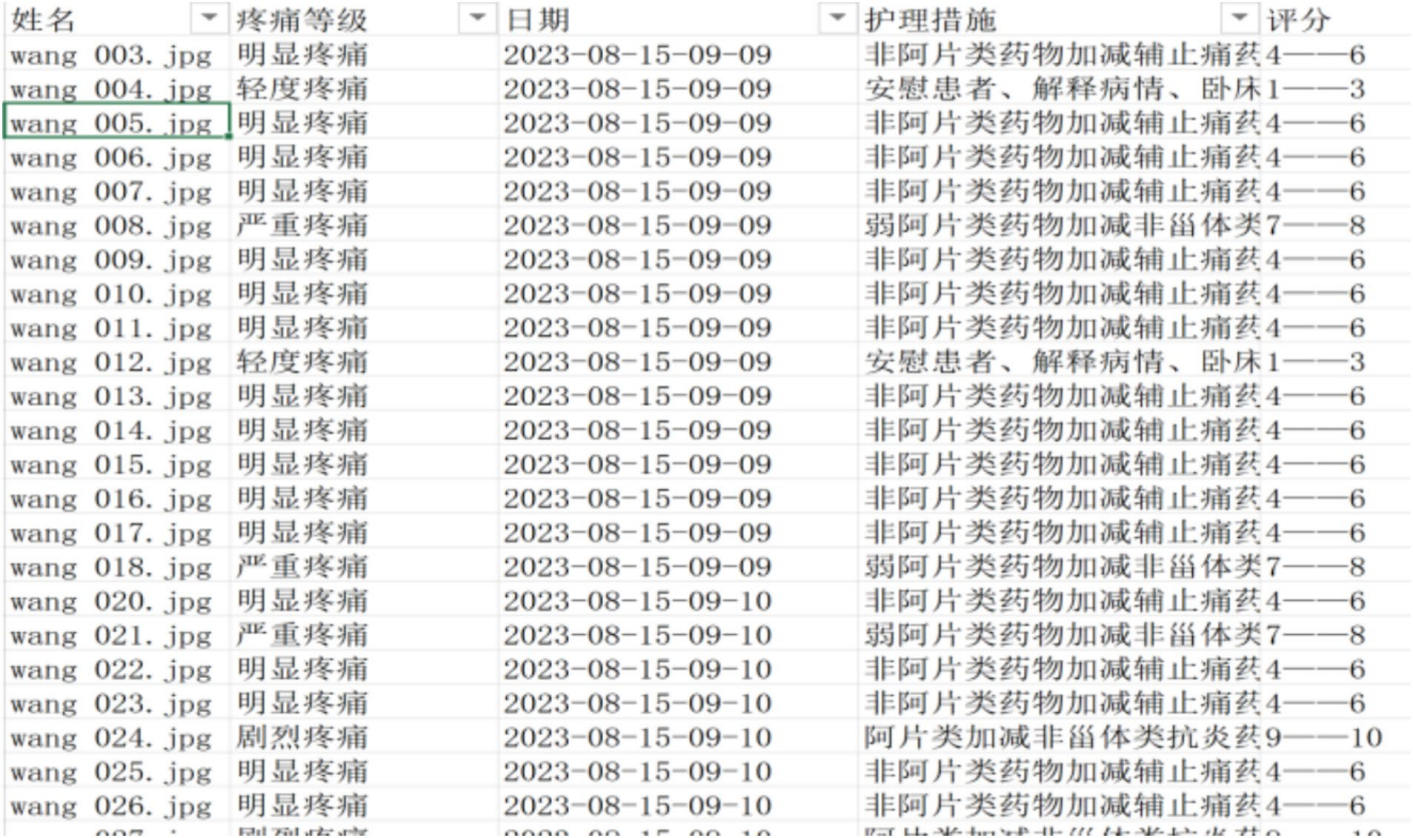
Patients’ pain assessment record sheet.

The pain grade of the facial expression is determined by taking the label category with the highest probability of the Softmax output. To avoid false detections and improve system stability, the detected label must reach a stable and continuous number of frames before it can be used as the output result of the pain grade determination. The collected video data is organized into a folder and transmitted to a dedicated computer, which is carried out the pain expression classification automatically in video detection mode. The pain grade report has been generated with the longest duration of pain expression in the video (Figures 5, 6).

### 3.3 Measure

The study involved 15 clinicians, each with more than 5 years of clinical pain assessment experience, who underwent a training session. The session covered the structure of the Pain Evaluation and Entitlement Act (PEEA) report and included three videos to demonstrate the process. The trainer, who was familiar with the outputs of PEEA, provided guidance on where to find relevant information in the graphical outputs, but did not interpret any videos or images to allow the clinicians to utilize their medical expertise.

During the session, the clinicians were instructed to rate the pain grade based on their visual inspection of the patients’ condition solely. To ensure accurate assessment and avoid reader fatigue, the clinicians were allowed to take an unlimited amount of time, until completing the assessments. And then the researchers assessed pain grades of the same videos by the PEEA system.

Agreement rates between clinicians and PEEA were assessed by intraclass correlation (ICC), which was assuming random effects for 15 clinicians. 95% confidence intervals was calculated according to the original derivations by Shrout and Fleiss (27). Standard errors of the mean for ICCs were estimated by resampling the observations with replacement (bootstrap) 1,000 times. The two-way of the same raters for all subjects has been selected in this study.

## 4 Results

### 4.1 PEEA performance

**Figure 7:**
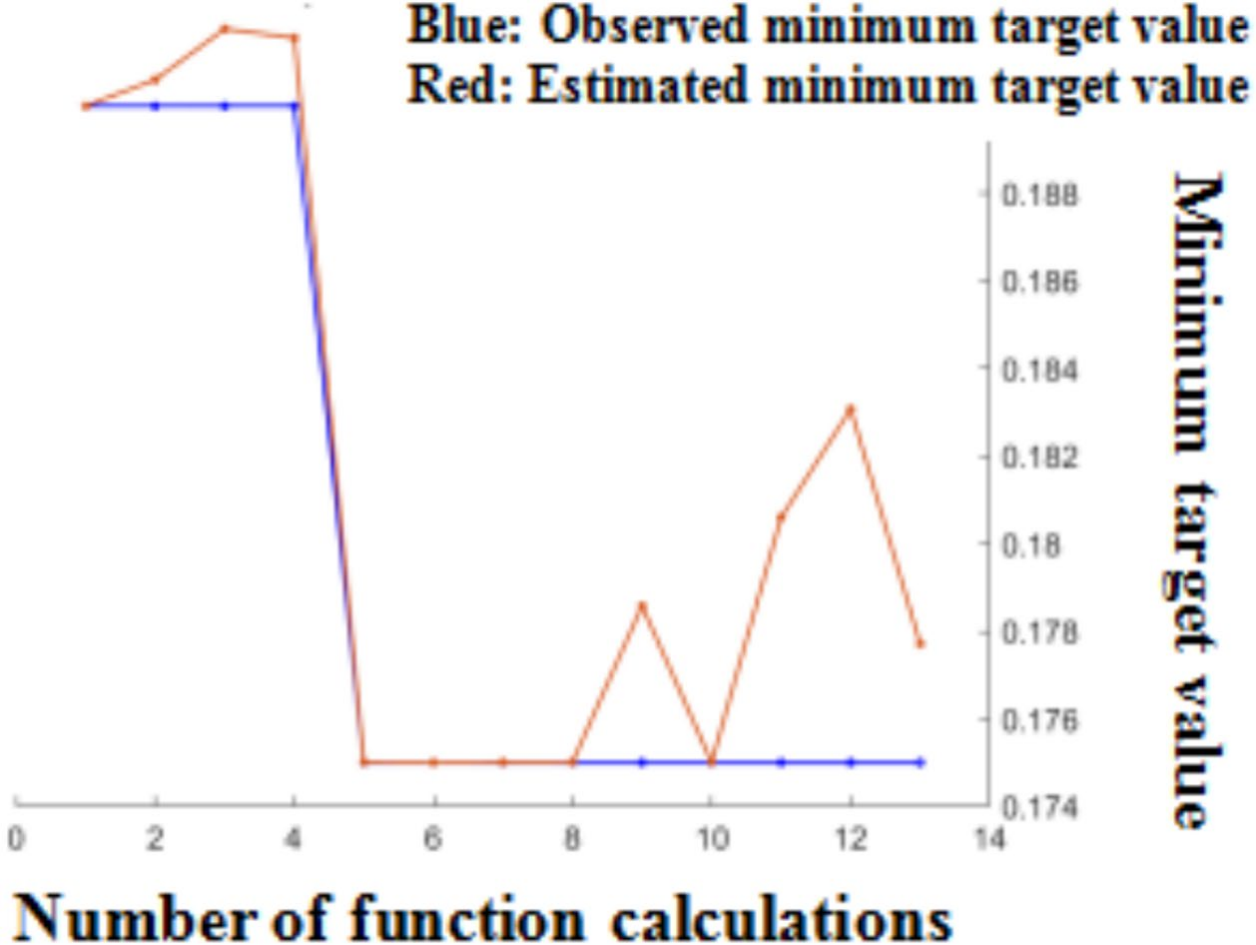
Minimum target value and number of function calculations.

Following Bayesian hyperparameter optimization, the learning rate, momentum, and L2 regularization were been determined, which were 0.1679, 0.8437, and 2.456 × 10–5, respectively. The optimizer utilized the SGDM optimization algorithm, which updated model parameters using a momentum factor while selecting training data samples. Comparing to the RMSProp and Adam optimizers, this method has better training speed and prediction accuracy (Figure 7).

The trained model has been tested in a clinical setting, and the results of the statistical data analysis have been presented in Table 3. The experimental analysis demonstrated that improved network has higher accuracy in both the training and validation sets, with better performance than the ResNet50 network for classification and recognition of pain expressions in elderly patients with hip fractures, which has higher training efficiency.

**Table 3:** Comparison of pain expression classification models in elderly hip fracture patients.

Network model	Alexnet	ResNet15	ResNet50	Improved network
Training set accuracy /(%)	77.4	85.6	98.4	99.6
Validation set accuracy /(%)	74.5	83.4	97.5	98.7
Number of iterations	50	50	50	30
Total training time / (min)	52	36	46	24

**Figure 8:**
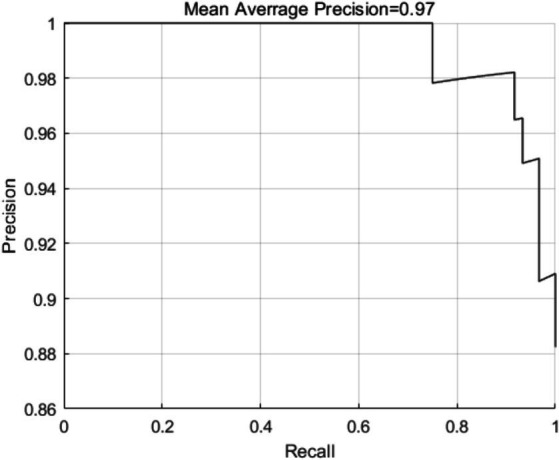
Schematic diagram of detection precision of the MTCNN model in pain expression recognition method for elderly individuals with hip fractures.

To assess the effectiveness of the MTCNN model for face detection in this study, we evaluated the detection precision and speed in face testing by 32 patients. As shown in Figure 8, the detection precision was 0.97, and the detection speed was 0.023 s/image when using a processor of Core (TM) i5-12400, indicating a high detection speed and precision for the proposed face detection algorithm. The model converged at 1063 iterations, and the result of final validation set was up to 98.7% approximately.

### 4.2 Validation

**Figure 9:**
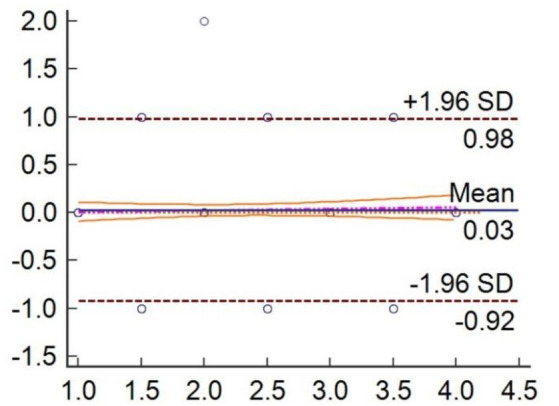
Individual agreement between clinician independently and PEEA for measure the pain videos.

For the validation analysis, we selected 300 pain videos of elderly patients with hip fractures, with 28 excluded due to dim lighting, resulting in 272 expression videos for 182 patients with hip features for analysis. The pain assessment and video capture are both conducted 24 and 48 h after the patient’s injury. Some participants collected two videos. The demographic and clinical characteristics of the enrolled patients are shown in Table 4. The pain scores between clinicians and PEEA were been compared. And the results demonstrated that PEEA has good reliability in pain assessment, with an average intraclass correlation measure of 0.9257 (Tables 5, 6) and a substantial kappa coefficient of 0.683 (Table 7; Figure 9).

**Table 4:** The participants characteristics (*N* = 182).

Characteristics	Mean ± SD or Number (%)
Age, years	77.35 ± 8.2
Sex, male	78
Ethnicity, Han nationality	173
Living status	
Living alone	105
Living with spouse or grown children	77
Education^a^	
Low	43
Medium	116
High	23
Main comorbidity	
Hypertension	132
Diabetes	81
Cardiac	143

^a^Low = none, primary school, lower-level vocational training, lower-level secondary general education; medium = middle-level vocational training, higher-level secondary general education; high = higher-level vocational training, academic education.

**Table 5:** The pain grade of videos between clinician and PEEA.

VAS	1–3	4–6	7–8	9–10
Clinician	68	90	91	23
PEEA	69	94	88	21

**Table 6:** Intraclass correlation coefficient between clinician and PEEA.

	Intraclass correlation^a^	95% Confidence interval
Single measures^b^	0.8616	0.8276 to 0.8894
Average measures^c^	0.9257	0.9056 to 0.9414

^a^The degree of consistency among measurements.^b^Estimates the reliability of single ratings.^c^Estimates the reliability of averages of k ratings.

**Table 7:** Symmetry measure of the pain videos.

		Value	Asymptotic standard error^a^	Approximation T^b^	*p*
Coherence measure	Kappa	0.683	0.036	17.976	0.000
Effective cases	N	272			

^a^No assumption of zero.^b^Assuming zero hypothesis using asymptotic standard error.

## 5 Discussion

The effects of data enhancement on model performance were evaluated by training the original and enhanced datasets under the same parameters and network structure. The results showed that the testing accuracy of the enhanced dataset was higher than that of the unexpanded original dataset, indicating that data enhancement can increase data diversity and prevent over-fitting during training. The increased training data also led to more detailed feature types, resulting in more stable training accuracy and reduced fluctuations, which improved the model’s robustness effectively.

The method for recognizing pain expressions in elderly patients with hip fractures involved acquiring a face image, using MTCNN for face detection and pre-processing, building and training a improved Resnet50 network model, and inputting images for pain grade prediction. By integrating MTCNN and Resnet50 network and performing transfer learning on a self-built dataset, the proposed model achieved accurate classification of pain expressions in elderly patients with hip fractures.

In this study, we constructed a deep learning network model for recognizing and classifying pain expressions in elderly patients with hip fractures. The residual module in the model addressed the problem of network degradation effectively, ensuring that performance did not decline with deepened network depth. To tackle the problem of small publicly labeled image datasets on pain expression, transfer learning has been used to avoid over-fitting.

The results showed that PEEA had high accuracy and efficiency, indicating that they tend to decrease nondeterminacy. This is important because nondeterminacy can lead to unnecessary interventions or examinations, costing time, money, and causing discomfort and anxiety for patients. The technology could allow clinicians to recognize pain grades more quickly. However, because our study only included 15 clinicians, we used the type of ICC that considers the clinician as a random effect to quantify agreement rates. This allows us to generalize the results to a broader population. This study strictly adhered to ethical principles during implementation, fulfilled the obligation of disclosure before the experiment, collected videos with the patient’s consent. This study mainly evaluated the dynamic pain grade of patients. The pain situation in the resting state was not be included in this study. Functional exercise and pain assessment are performed on patients in an independent space relatively to reduce external interference factors and protect patient privacy. At the same time, the database of the pain automatic classification systems stored in the specific computer folder. Protective measures has been set up to prevent arbitrary copying and leakage of patient information.

Artificial intelligence has the potential to revolutionize nursing and medicine. Our study highlighted that these software systems are meant to support and enhance clinicians’ performance in clinical practice rather than replace them. Automated assessment systems, such as PEEA, which could be used to assess and grade a large number of pain videos or images quickly, improving the reliability of measurements by decreasing interobserver variability.

The limitations of this study are that: (1) The results may lack effective persuasion because the number of samples is limited, especially in model validation. (2) The evidence of criterion-related validity was limited and sensitivity has not been measured. Although the positioning accuracy of model must be sufficient, but this model has targeted clearly, so the number of samples can be reduced. (3) Pain is a subjective feeling that is influenced by many factors, such as personality, social environment, previous pain experiences, etc. At present this study cannot exclude all influencing factors to accurately evaluate pain grades. In future research, specific character models can be established to improve pain expression recognition results. (4) This study is based on facial expression recognition of pain grades in elderly patients, with limited information. In future research, we should pay more attention to the means of multi information fusion to further improve the accuracy of pain expression recognition.

## 6 Conclusion

Traditional convolutional neural network (CNN) has been used for recognizing and classifying pain expression in elderly patients with hip fractures suffers from long training times and poor accuracy. In this study, we improved the hyperparameters of the ResNet50 network using Bayesian optimization. The results confirmed that this network can construct an automatic pain expression recognition and classification model for elderly patients with hip fractures. Comparing experimental analysis, the study led to the following conclusions: The automatic pain expression recognition model for elderly patients with hip fractures has been constructed based on the algorithm of this study which does not require manual extraction of facial features, reducing reliance on experience and expertise. The improved network has higher accuracy, fewer training runs and shorter training time.

In conclusion, our study suggests that computer-assisted detection systems, such as PEEA, which can improve both the speed and efficiency of pain grade assessments. This technology can be extended to the clinical evaluation of elderly people aged 65 and above, especially those with traumatic fractures. However, due to limitations in database data, the effect of this method in young patients with fractures still needs further research.

## Data availability statement

The raw data supporting the conclusions of this article will be made available by the authors, without undue reservation.

## Ethics statement

The studies involving humans were approved by Tianjin Hospital Medical Ethics Committee, Tianjin Hospital. The studies were conducted in accordance with the local legislation and institutional requirements. The participants provided their written informed consent to participate in this study. Written informed consent was obtained from the individuals for the publication of any potentially identifiable images or data included in this article.

## Author contributions

YS: Formal analysis, Supervision, Writing – review & editing. GL: Methodology, Validation, Writing – original draft. ZH: Funding acquisition, Investigation, Writing – original draft. LJ: Data curation, Formal analysis, Investigation, Project administration, Writing – review & editing. CX: Data curation, Formal analysis, Investigation, Writing – original draft. SS: Investigation, Writing – original draft. ZX: Investigation, Writing – original draft. LW: Methodology, Supervision, Writing – review & editing.
